# Prediction of Central Nervous System Relapse of Diffuse Large B-Cell Lymphoma Using Pretherapeutic [^18^F]2-Fluoro-2-Deoxyglucose (FDG) Positron Emission Tomography/Computed Tomography

**DOI:** 10.1097/MD.0000000000001978

**Published:** 2015-11-06

**Authors:** Yoo Sung Song, Won Woo Lee, Jong Seok Lee, Sang Eun Kim

**Affiliations:** From the Department of Nuclear Medicine (YSS, WWL, SEK); and Department of Internal Medicine, Seoul National University Bundang Hospital, Seoul, South Korea (JSL).

## Abstract

Central nervous system (CNS) relapse of diffuse large B-cell lymphoma (DLBCL) is a rare complication, but has a poor prognosis with unknown pathophysiology. Recent trials of CNS prophylaxis have shown to be ineffective, despite patient's selection using several known clinical risk factors. In this study, the authors evaluated the value of pretreatment [^18^F]2-Fluoro-2-deoxyglucose positron emission tomography in predicting CNS relapse in DLBCL patients.

The authors analyzed 180 pathologically confirmed DLBCL patients, retrospectively. Patients underwent [^18^F]2-Fluoro-2-deoxyglucose positron emission tomography/computed tomography before first line rituximab to cyclophosphamide, doxorubicin, vincristine, and prednisone therapy. Clinical characteristics were evaluated and total lesion glycolysis (TLG) with a threshold margin of 50% was calculated.

Among age, sex, Ann Arbor stage, International Prognostic Index, revised International Prognostic Index, high serum lactate dehydrogenase level, presence of B symptoms, bulky disease (≥10 cm), extranodal lesion involvement, bone marrow involvement, high metabolic tumor volume ( >450 mL), and high TLG50 (>2000), the high TLG50 was the only significant prognostic factor for predicting CNS relapse in a multivariate analysis (*P* = 0.04). Kaplan–Meir survival analysis between high TLG50 (>2000) and low TLG50 (≤2000) groups revealed significantly different mean progression free survival (PFS) of 1317.2 ± 134.3 days and 1968.6 ± 18.3 days, respectively (*P* < 0.001).

High TLG50 on [^18^F]2-Fluoro-2-deoxyglucose positron emission tomography/computed tomography is the most significant predictor of CNS relapse in un-treated DLBCL patients.

## INTRODUCTION

Relapse of diffuse large B-cell lymphoma (DLBCL) in the central nervous system (CNS) is a rare but serious complication. It has been reported to occur in 2% to 8% of patients during the course of DLBCL.^1,2^ Central nervous system relapse occurs in the early course of DLBCL, with a short median time to recurrence (5–12 months).^3^ Also, patients once diagnosed with CNS relapse have a short median survival of 2 to 5 months.^1,4,5^ Thus, the fatal prognosis of CNS relapse has highlighted the importance of identifying patients at high risk to provide prophylactic therapy as a preventative approach.

Numerous reports have suggested various risk factors for high risk of CNS relapse, and they are high serum lactate dehydrogenase level, International Prognostic Index (IPI), and involvement of specific extranodal organs.^5–8^ Patient selection criteria for CNS prophylaxis in previous studies were based on these risk factors, in pursuit of lowering the incidence of CNS relapse. Despite the low incidence of CNS relapse, the effect of CNS prophylaxis with intrathecal methotrexate (IT-MTX) on aggressive lymphoma has been studied in numerous reports. Most studies, however, revealed that IT-MTX did not lower the incidence of CNS relapse.^6,9–13^ The Southwest Oncology Group 8516 study, a prospective randomized trial with 20-year follow-up showed that IT-MTX added no additional benefits to CNS relapse free survival.^6^ Another multicenter international clinical trial with 2210 patients revealed that the addition of rituximab to cyclophosphamide, doxorubicin, vincristine, and prednisone (R-CHOP) reduced CNS prophylaxis compared with CHOP, but IT-MTX prophylaxis did not.^9^ Studies confined to DLBCL patients receiving R-CHOP also revealed the ineffectiveness of IT-MTX prophylaxis.^10^ Thus, despite the numerous studies, no definite criteria are accepted and widely varying combinations of the above factors are used currently among clinicians.^1,14^ This lack of guidelines results in inconsistency of CNS prophylaxis protocols and unwanted toxicity to patients because of overtreatment,^15,16^ and therefore, requires a more concrete selection criterion.

[^18^F]2-Fluoro-2-deoxyglucose (FDG) positron emission tomography (^18^F-FDG PET) is a noninvasive imaging method widely used in the management of malignant lymphoma.^17–19^ A recent study has suggested total lesion glycolysis (TLG) as an efficient prognostic index of DLBCL, which is calculated by mean standard uptake value (meanSUV) × metabolic tumor volume (MTV).^20^ In this study, we evaluated the significance of TLG regarding the prediction of CNS relapse in the chemotherapy-naïve DLBCL patients.

## PATIENTS AND METHODS

### Patients

A total of 180 newly diagnosed DLBCL patients between March 2009 and January 2015 at Seoul National University Bundang Hospital, South Korea, were retrospectively enrolled. This study was approved by the Institutional Review Board for review of medical records of the patients and was performed in accordance with the ethical standards laid down in the 1964 Declaration of Helsinki and its later amendments. For this type of study, formal consent is not required and the acquisition of informed consents was exempted. Inclusion criteria were patients with pathologic confirmation of DLBCL, first line therapy of R-CHOP, and ^18^F-FDG PET/computed tomography (CT) before treatment. Exclusion criteria were patients who had surgical excision of DLBCL main lesion before ^18^F-FDG PET/CT, and primary CNS lymphoma. The following clinical data were obtained: age, sex, Ann Arbor stage, Eastern Cooperative Oncology Group performance score , IPI,^21^ revised IPI (R-IPI),^22^ pretreatment serum lactate dehydrogenase (LDH) level, presence of B symptoms, bulky disease (≥10 cm), extranodal involvement, and bone marrow involvement.

### Treatment and Follow-Up

Patients were treated with standard R-CHOP chemotherapy with or without radiation therapy, with a treatment interval of 3 weeks. Ann Arbor stage I/II patients received 4 to 6 cycles, and stage III/IV patients received 6 to8 cycles of R-CHOP chemotherapy. Central nervous system prophylaxis with IT-MTX was given in patients with high intermediate/high IPI risk or extranodal involvement of testis/breast/nasal cavity/orbit. Follow-up examination for CNS relapse was done on a regular outpatient basis or when the patient had suspicious symptoms, using cerebrospinal fluid (CSF) cytology examination or brain magnetic resonance imaging (MRI).

### [^18^F]2-Fluoro-2-deoxyglucose Positron Emission Tomography/Computed Tomography

^18^F-FDG PET/CT images were obtained before the start of R-CHOP, using a PET/CT scanner (Discovery VCT, GE Medical Systems, Milwaukee, WI). Patients were fasted for at least 6 hours, and 5.18 MBq/Kg of ^18^F-FDG were injected. Images were obtained 40 minutes after ^18^F-FDG injection. Computed tomography images were acquired first from the base of cerebellum to upper thigh (120 kVp, 3.75 mm slice thickness). Positron emission tomography images were acquired in a three-dimensional acquisition mode (5–6 bed position, 2.5 minutes/bed). Positron emission tomography images were reconstructed on a 128 × 128 matrices using an iterative algorithm (ordered subset expectation maximization, 2 iterations and 8 subsets), with CT-based attenuation correction. For image analysis, SUV was calculated as [decay-corrected activity (kBq) per mL of tissue/injected activity (kBq) per lean body mass (g)]. Metabolic tumor volume with a threshold margin of 50% of maxSUV was used. Total lesion glycolysis was calculated as (meanSUV × MTV). Threshold margin of 50% of maxSUV was adapted from a previous study, in which high TLG50 was significantly associated with poor overall prognosis of DLBCL patients.^20^ Standard uptake value, MTV, and TLG values were measured using an analysis software package (Syngo via, Siemens Healthcare, Germany).

### Statistical Analysis

Factors between subgroups of patients were evaluated with χ^2^ test, or Fisher exact test. Endpoint was progression free survival (PFS), from the start of treatment to CNS relapse. Independent risk factors associated with CNS relapse were evaluated with univariate/multivariate Cox proportional hazards regression model. Progression free survival curves were derived from Kaplan–Meier survival analysis. A commercial software package (MedCalc, Version 12.2.1.0, MedCalc Software, Belgium) was used for the analyses. *P* values less than 0.05 were regarded as significant.

## RESULTS

### Patient Characteristics

Clinical characteristics of the 180 patients are shown in Table 1. The median age was 63 years (average 61.2 ± 13.6), with 104 men and 76 women. Overall PFS was 1825.2 ± 46.3 (95% confidence interval, CI = 1734.5–1916.0) days, with CNS relapse in 12 patients (6.7%). The pattern of CNS relapse was intracerebral in 6 patients, and leptomeningeal seeding in 6 patients.

**TABLE 1:**
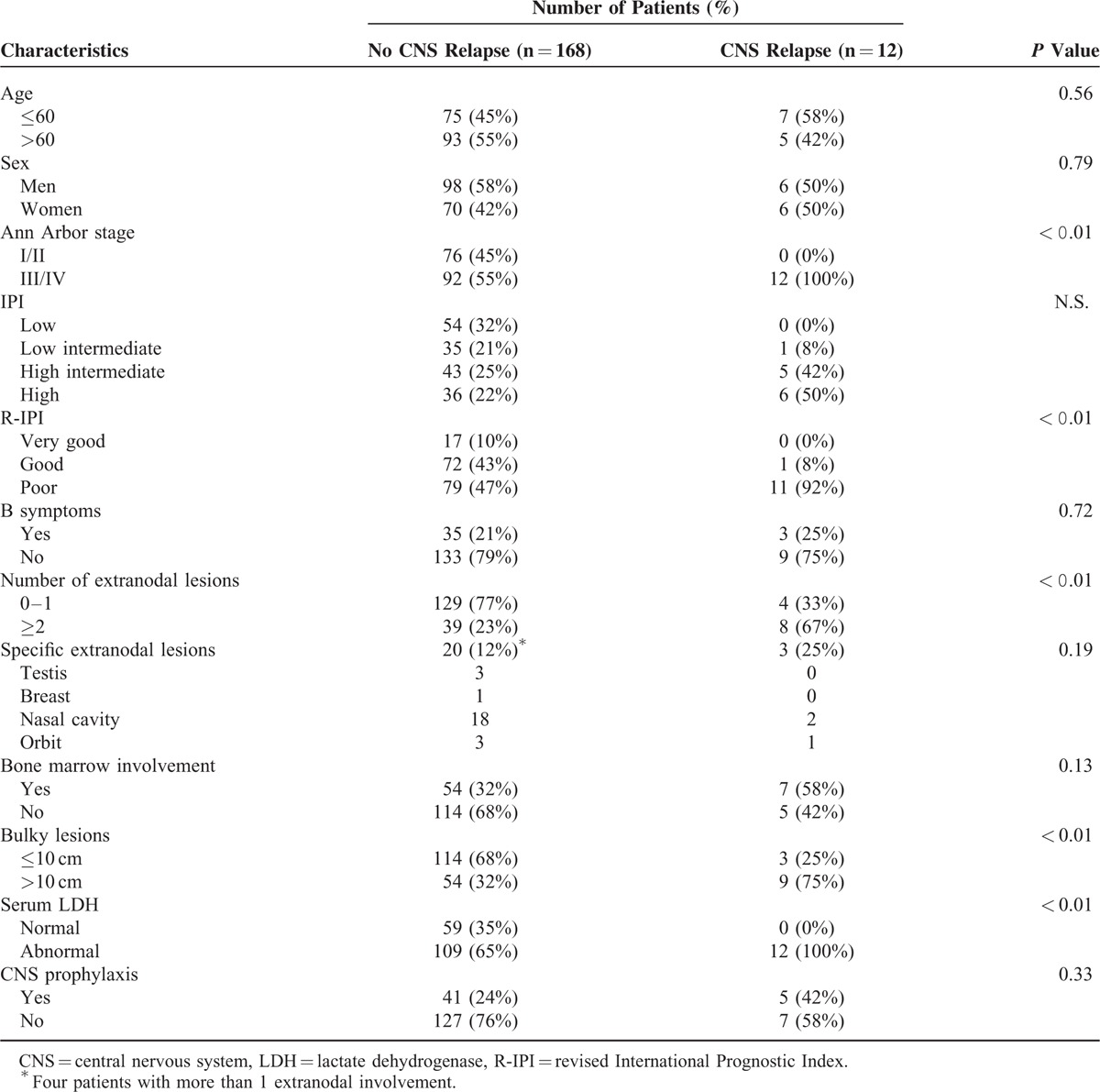
Clinical Characteristics of Patients

### Risk Factors for Central Nervous Systems Relapse

Patient group was divided into those above and below 2000 in terms of TLG50, and above and below 450 (mL) in terms of MTV. Cutoff values of 2000 for TLG50 and 450 (mL) for MTV were the most significant values for predicting CNS relapse, obtained from the receiver operating characteristic curve. The sensitivity and specificity were 92%, 99% for TLG50 of 2000, and 75%, 82% for MTV of 450 (mL), respectively. By univariate analysis, high IPI (*P* < 0.01, hazard ratio, HR = 2.75, 95% CI = 1.40–5.40), high R-IPI (*P* = 0.02, HR = 11.45, 95% CI = 1.55–85.77), presence of a bulky lesion (*P* < 0.01, HR = 7.20, 95% CI = 1.95–26.64), involvement of bone marrow (*P* = 0.03, HR = 3.61, 95% CI = 1.14–11.49), high MTV (>450) (*P* < 0.001, HR = 9.42, 95% CI = 2.83–31.30), and high TLG50 (>2000) (*P* < 0.001, HR = 33.91, 95% CI = 4.42–260.49) were significant risk factors for CNS relapse (Table 2). Central nervous system prophylaxis did not reduce the risk of CNS relapse (*P* = 0.15), in concordance with previous reports. By multivariate analysis, high TLG50 (>2000) (*P* = 0.04, HR = 11.99, 95% CI = 1.06–135.42) was the only significant risk factor for CNS relapse (Table 3).

**TABLE 2:**
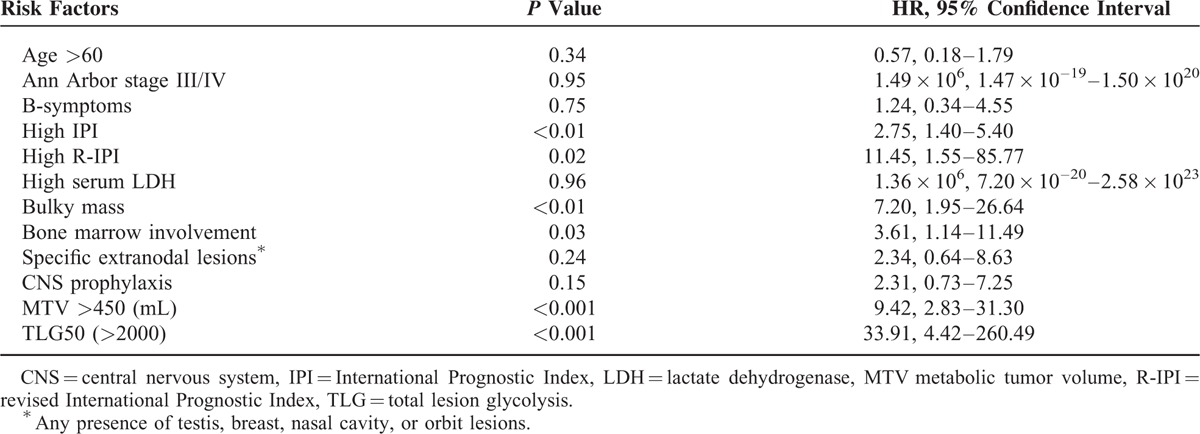
Univariate Analysis of Risk Factors for Central Nervous System Relapse

**TABLE 3:**
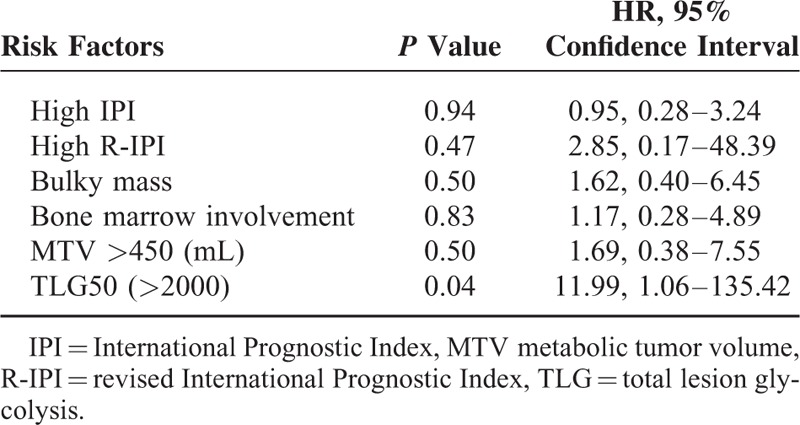
Multivariate Analysis of Risk Factors for Central Nervous System Relapse

### Progression Free Survival Analysis of Central Nervous System Relapse

**FIGURE 1:**
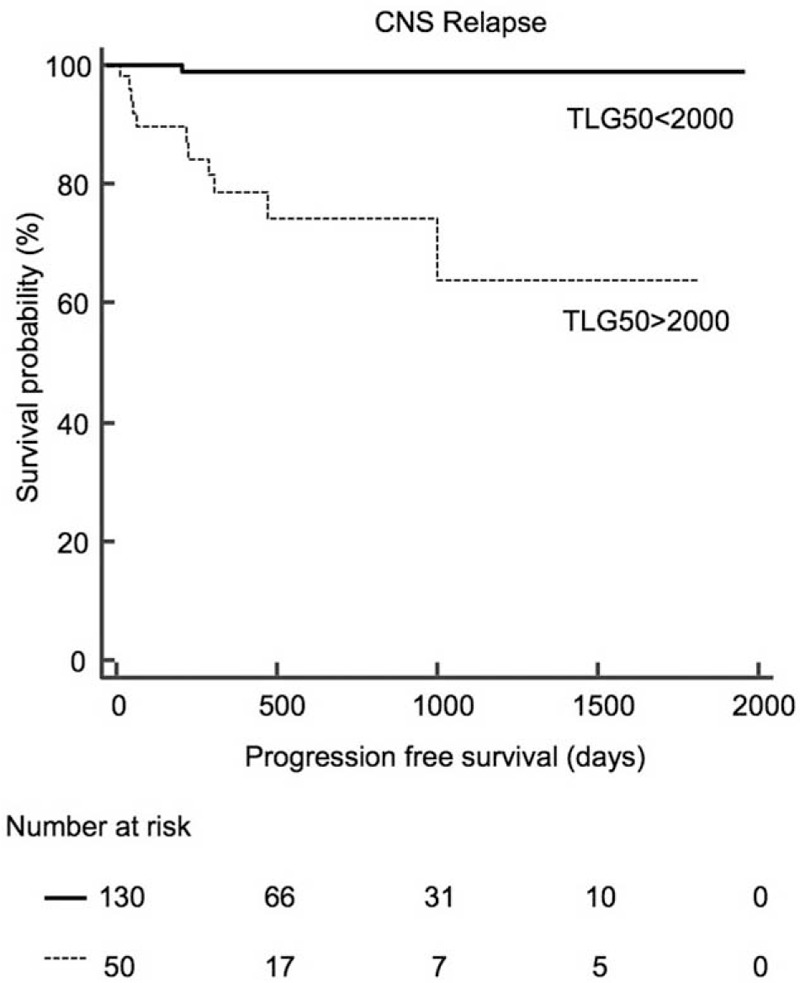
Kaplan–Meier survival curves for progression free survival of central nervous system relapse, according to total lesion glycolysis50.

**FIGURE 2:**
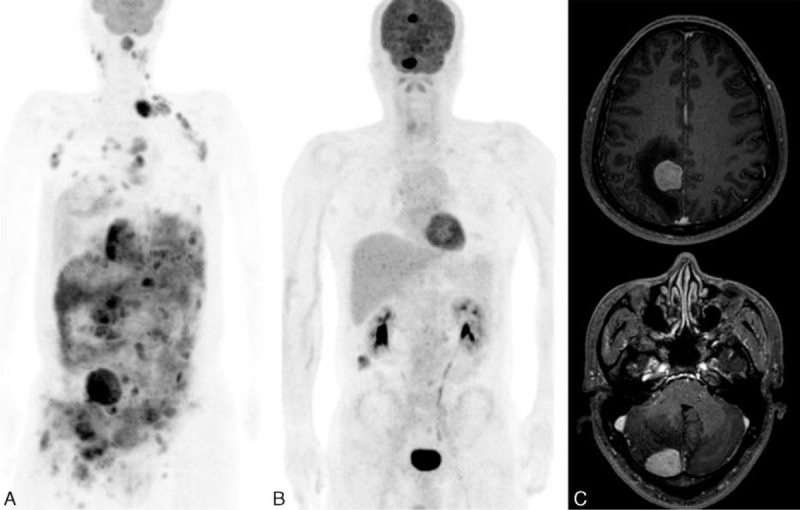
Representative case of a patient with total lesion glycolysis50 (>2000). A 52-year-old patient was diagnosed Ann Arbor stage III diffuse large B-cell lymphoma, with multiple lymphoma lesions. Serum lactate dehydrogenase was 667 IU/L. The patient did not have lymphoma involvement in the bone marrow, testis, nasal cavity, or orbit. A, Total lesion glycolysis was measured as 5280.5 on initial [^18^F]2-Fluoro-2-deoxyglucose positron emission tomography/computed tomography. B, Follow-up [^18^F]2-Fluoro-2-deoxyglucose positron emission tomography/computed tomography was performed 7 months later and new intracerebral diffuse large B-cell lymphoma lesions were found in the right parietal lobe and right cerebellum. C, magnetic resonance imaging reveals homogenous enhancement of the lesions.

**FIGURE 3:**
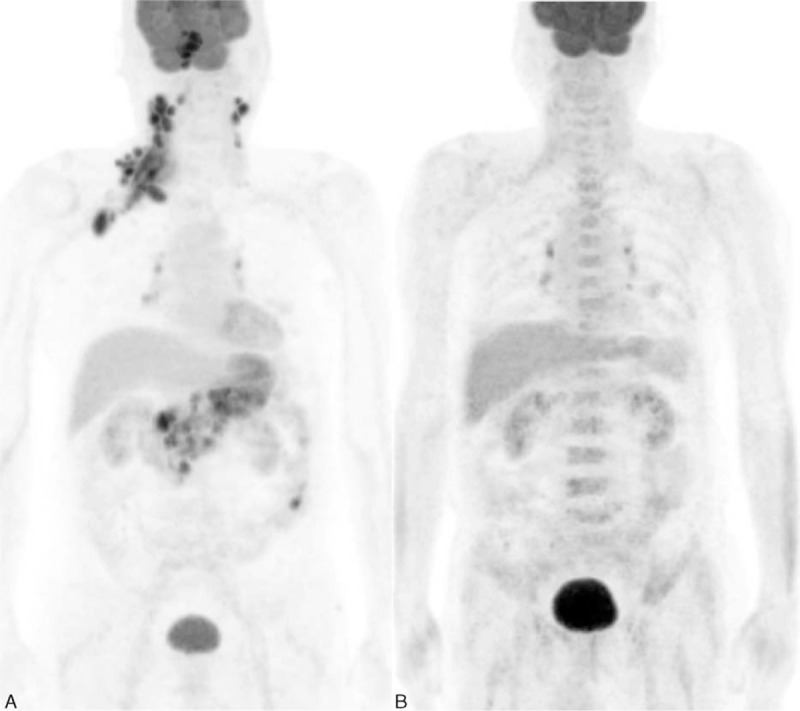
Representative case of a patient with total lesion glycolysis50(<2000). An 83-year-old patient was diagnosed Ann Arbor stage III diffuse large B-cell lymphoma. Serum lactate dehydrogenase was 390 IU/L. A, The patient had lymphoma involvement in the nasal cavity, and total lesion glycolysis50 was measured as 1142.9 on ^18^F-FDG PET/CT. B, Lesions disappeared on follow-up PET/CT, without central nervous system relapse.

When patients were classified by TLG50 (≤2000) and TLG50 (>2000), the 2 groups exhibited a significant PFS difference in Kaplan–Meier survival analysis (*P* < 0.001, HR = 33.41, 95% CI = 9.01–123.89) (Figure 1). Among the patient group with TLG50 >2000 (n = 50), mean PFS was 1317.2 ± 134.3 (95% CI = 1054.0–1580.4) days and CNS relapse occurred in 11 patients (22%). On the contrary, among patient group with TLG50 (≤2000) (n = 130), mean PFS was 1968.6 ± 18.3 (95% CI = 1932.7–2004.5) days and CNS relapse occurred in only 1 patient (0.8%). The different patient characteristics between patient groups with TLG50 (≤2000) and TLG50 (>2000) are listed in Table 4. Ann Arbor stage III/IV (*P* < 0.001), IPI (*P* < 0.001), R-IPI (*P* < 0.001), presence of B symptoms (*P* = 0.04), presence of more than 1 extranodal lesions (*P* < 0.001), presence of bulky lesions (*P* < 0.001), bone marrow involvement (*P* < 0.01), and high serum LDH level (*P* < 0.001) were significantly different between the 2 groups. Representative cases of each group are illustrated in Figure 2 and Figure 3.

**TABLE 4:**
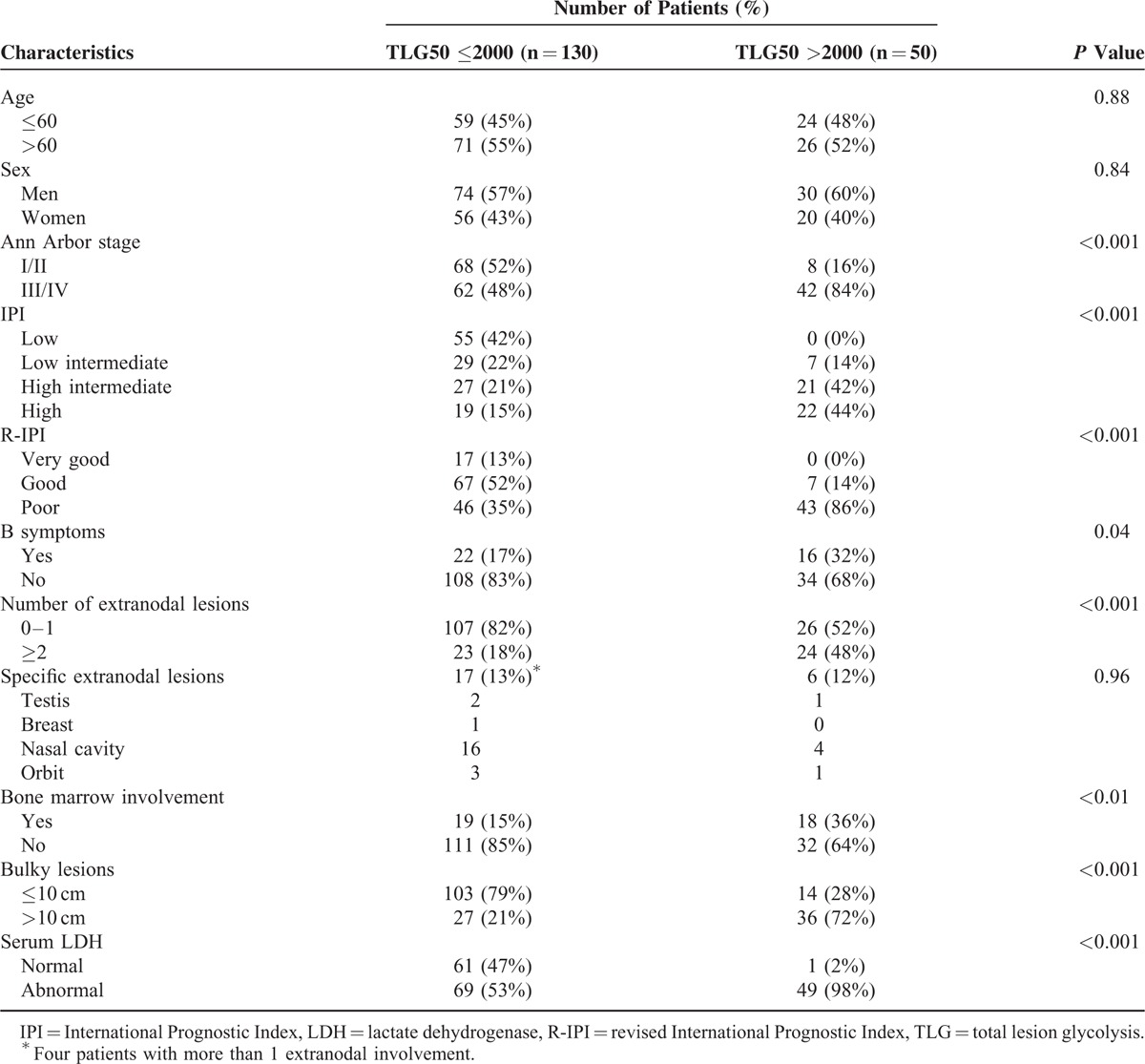
Patient Characteristics According to Total Lesion Glycolysis50

## DISCUSSION

In this study, we demonstrated that TLG50 is a significant prognostic factor of CNS relapse in DLBCL patients. TLG50 (>2000) was the single significant risk factor among currently used clinical indicators, such as Ann Arbor stage, IPI, R-IPI, LDH, and presence of extranodal involvement. The value of TLG in evaluating prognosis has been studied widely in other types of cancer, such as non-small cell lung cancer, head and neck cancer, pancreatic cancer, and gynecologic cancer.^23–26^ There are several explanations for TLG being a good indicator for prognosis. Conventional tumor-node-metastasis staging or Ann Arbor staging does not quantitatively reflect the tumor burden or histopathologic aggressiveness, especially in the advanced status. Also as our results indicate, MTV was not a significant indicator in multivariate analysis. This could be because of the fact that though MTV reflects the tumor burden, histopathologic aggressiveness is not considered. Because DLBCL is known to have a different prognostic behavior according to its molecular profile,^27^ histopathologic consideration would be important. TLG, which is a factor derived from the combination of MTV and SUV, however, is more apt to predict patients’ prognosis because MTV represents the tumor burden and SUV reflects the tumor aggressiveness, respectively.

Previous studies defined patients with high risk of CNS relapse with a criteria in combination of specific extranodal involvement, bone marrow involvement, and elevated serum LDH,^4,11,13,28^ but the incidences of CNS relapses have been reported to be similar within high-risk and low-risk patient groups, ranging from 2% to 10%. Thus, we suggest that our selection criteria of high-risk CNS relapse based on TLG50 may have several advantages over the conventional patient selection criteria. First, using high TLG50 may reduce unwanted treatment related toxicity. CNS prevention regimens are based on IT-MTX, with several concomitant administration of intravenous MTX.^29^ As MTX is known for several severe toxicities such as paralysis, seizure, cranial nerve palsy, and myelosuppression,^30,31^ cutting down the target population for CNS prophylaxis is important in terms of reducing treatment related morbidity. In a study defining high-risk patients with extranodal site involvement or elevated serum LDH with advanced stage, 54% of the whole patients were classified as high-risk group and received CNS prophylaxis. Only 8% of the high-risk patients, however, developed CNS relapse.^32^ In comparison, only 28% (50 of 180 patients) of the whole patients were classified as high risk with high TLG50 (>2000) whereas 22% of high-risk patients developed CNS relapse in our study. Secondly, high TLG50 may be used as a simplified selection criterion for high-risk patients. Physicians currently perform CNS prophylaxis in high-risk patient group with combination of clinical risk factors, with or without cerebrospinal fluid (CSF) cytology/flow cytometry under their own policy. Screening with CSF examination in every patient of high-risk group, however, may not be cost-effective. And this screening method has a low sensitivity for CNS relapse, because it eventually leaves out approximately 50% of all CNS relapses, which are in the low-risk group.^14^ Additive effect of conventional neuroimaging methods is also questionable, because of its low sensitivity. Although CT imaging is known to have less sensitivity compared with MRI,^33^ MRI itself has a high false-negative rate of >30%.^34^ Our study detected CNS relapse with a sensitivity of 92% (11 of 12 patients). Moreover, clinical factors that were significantly different between patient groups with and without relapse also showed significant differences between patient groups with high and low TLG50 (Ann Arbor stage, R-IPI, presence of more than 1 extranodal lesions, presence of bulky lesions, and high serum LDH). These findings suggest that high TLG50 may be a feasible simplified indicator in clinical practice.

The mechanism of CNS relapse may be explained by preexisting occult lymphoma cells in the CNS. Recent studies with CSF cytology/flow cytometry support the presence of occult lymphoma cells in CNS in the early stage of disease.^35–37^ Patients with extranodal involvement or high serum LDH were classified as high risk for CNS relapse, and underwent CSF examination. Occult lymphoma cells were found in 10% to 22% of patients. This is also supported by the fact that CNS relapse occurs in the early course of treatment, and that first line R-CHOP poorly penetrates the blood-brain barrier.^8^ In general, leptomeningeal seeding of malignant cells is associated with the aggressiveness and extent of systemic dissemination of primary tumors.^34^ Because TLG is an indicator that reflects both tumor aggressiveness and tumor burden as previously mentioned, patients with higher TLG are more likely to develop CNS relapse. In addition, 9 of 12 (75%) patients were free of systemic progression before developing CNS relapse. A total of six patients (50%) even reached metabolic complete remission status during the course of treatment. Thus, our report supports the existence of occult lymphoma cells before treatment, and initial evaluation with TLG can be an effective tool in predicting CNS relapse.

There, however, are several limitations in our study. First, this study design is retrospective. Second, although patients received R-CHOP as a first line chemotherapy, patients with disease progression or who have not reached complete remission status received heterogeneous second line chemotherapy regimens. Finally, there may exist some selection bias for CNS prophylaxis among physicians, which could not be properly controlled by the retrospective review of the medical record.

In summary, our study demonstrates that high TLG50 can be used as an indicator for predicting CNS relapse in DLBCL patients. It was a single significant prognostic factor amongst previous known clinical risk factors such as high serum LDH, presence of extranodal involvement, and bone marrow involvement. Further, prospective studies for evaluating the effect of CNS prophylaxis on high-risk CNS relapse patients according to TLG50 would be needed.
