# Prediction of Central Nervous System Relapse of Diffuse Large B-Cell Lymphoma Using Pretherapeutic [^18^F]2-Fluoro-2-Deoxyglucose (FDG) Positron Emission Tomography/Computed Tomography: Erratum

**DOI:** 10.1097/01.md.0000481353.63355.f4

**Published:** 2016-03-03

In the article “Prediction of Central Nervous System Relapse of Diffuse Large B-Cell Lymphoma Using Pretherapeutic [^18^F]2-Fluoro-2-Deoxyglucose (FDG) Positron Emission Tomography/Computed Tomography”,^1^ which appeared in Volume 94, Issue 44 of *Medicine*, the specificity value of 99% should have read 77% in the “Risk Factors for Central Nervous System Relapse” section. The article has since been corrected online.
